# Iodine-125 brachytherapy treatment for newly diagnosed brain metastasis in non-small cell lung cancer: A biocentric analysis

**DOI:** 10.3389/fonc.2022.1005876

**Published:** 2022-12-15

**Authors:** Lili Yang, Congxiao Wang, Wei Zhang, Shifeng Liu, Tiantian Xuan, Han Jiang, Xiaokun Hu, Man Hu, Huanting Li

**Affiliations:** ^1^ Department of the Interventional Medical Center, The Affiliated Hospital of Qingdao University, Qingdao, Shandong, China; ^2^ Department of Oncology, Qilu Hospital, Qingdao, Shandong, China; ^3^ Department of Radiation Oncology, Shandong Cancer Hospital and Institute, Shandong First Medical University and Shandong Academy of Medical Sciences, Jinan, Shandong, China; ^4^ Department of Neurosurgery, The Affiliated Hospital of Qingdao University, Qingdao, Shandong, China

**Keywords:** iodine-125, brachytherapy, brain metastasis, non-small cell lung cancer, external beam radiotherapy

## Abstract

**Purpose:**

The aim of the present study is to evaluate the safety and efficacy of iodine-125 brachytherapy for newly diagnosed brain metastasis in patients with non-small cell lung cancer (NSCLC).

**Materials and methods:**

The study included 158 NSCLC patients diagnosed with brain metastasis from December 2003 to August 2017. Ninety-nine patients underwent external beam radiotherapy (EBRT group), and 59 patients received iodine-125 brachytherapy (^125^I group). In addition, the 6- and 12-month progression-free survival (PFS) rates and the 12- and 24-month overall survival (OS) rates were compared between the EBRT group and the ^125^I group. Median OS and PFS were analyzed using the Kaplan−Meier method with a log-rank test.

**Results:**

The 6-month PFS rate was significantly higher in the ^125^I group (*p* = 0.002) than in the EBRT group, while no differences were found in the 12-month PFS rate (*p* = 0.184). Additionally, the 12- (*p* = 0.839) and 24-month (*p* = 0.284) OS rates were not significantly different between the two groups. No significant differences in median OS (*p* = 0.525) or PFS (*p* = 0.425) were found between the two groups.

**Conclusions:**

Iodine-125 brachytherapy is an alternative therapy for patients unable to undergo surgical resection.

## Introduction

Lung cancer is one of the leading causes of cancer-related mortality. Approximately 57% of patients with non-small cell lung cancer (NSCLC) present with metastasis. At the time of diagnosis, 20% of patients have brain metastases (BMs) (1, 2). Approximately 25% to 50% of patients will present with BMs during the course of the disease (3).

For BMs, surgical resection is often the option to alleviate symptoms. Various studies have confirmed the efficacy of surgical resection combined with postoperative radiation therapy (4). However, some of the patients presenting with BMs could not undergo surgical resection because of location, tumor volume, or poor medical conditions. Additionally, some patients reject the surgery and fear the side effects. The efficacy of radiotherapy without surgical resection has been approved. However, the prescribed dose of external beam radiotherapy (EBRT) should not be further increased in consideration of the safety of the surrounding normal tissues.

In contrast to primary brain tumors, infiltration of metastases seldom occurs in the brain. Based on these characteristics, local treatment was superior in controlling metastases (3). Numerous data confirmed the efficacy of ^125^I brachytherapy for local control. The minimally invasive, precise therapy allows a higher prescribed dose within the tumor and continuously releases low-dose rate γ-rays, which is different from EBRT. Various studies have confirmed the safety and efficacy of ^125^I brachytherapy for a variety of tumors (5). In the present study, we aimed to evaluate the safety and efficacy of ^125^I brachytherapy for newly diagnosed BMs in patients with NSCLC.

## Materials and methods

### Patient selection

The present study was approved by the institutional review boards of the two centers. The requirement for informed consent of the patients was waived. Patients’ data between December 2003 and 25 August 2017 were analyzed. The inclusion criteria were as follows: a) NSCLC patients diagnosed with BMs, b) patients who previously received systemic treatment, and c) patients who received ^125^I brachytherapy or EBRT as their initial treatment for BMs. The exclusion criteria were as follows: a) the BMs involved the bilateral cerebral hemisphere, b) the number of BMs was more than three, and c) patients with intratumoral hemorrhage. All patients could continue systemic treatment after EBRT or ^125^I implantation.

### Implantation of ^125^I seeds

**Figure 1:**
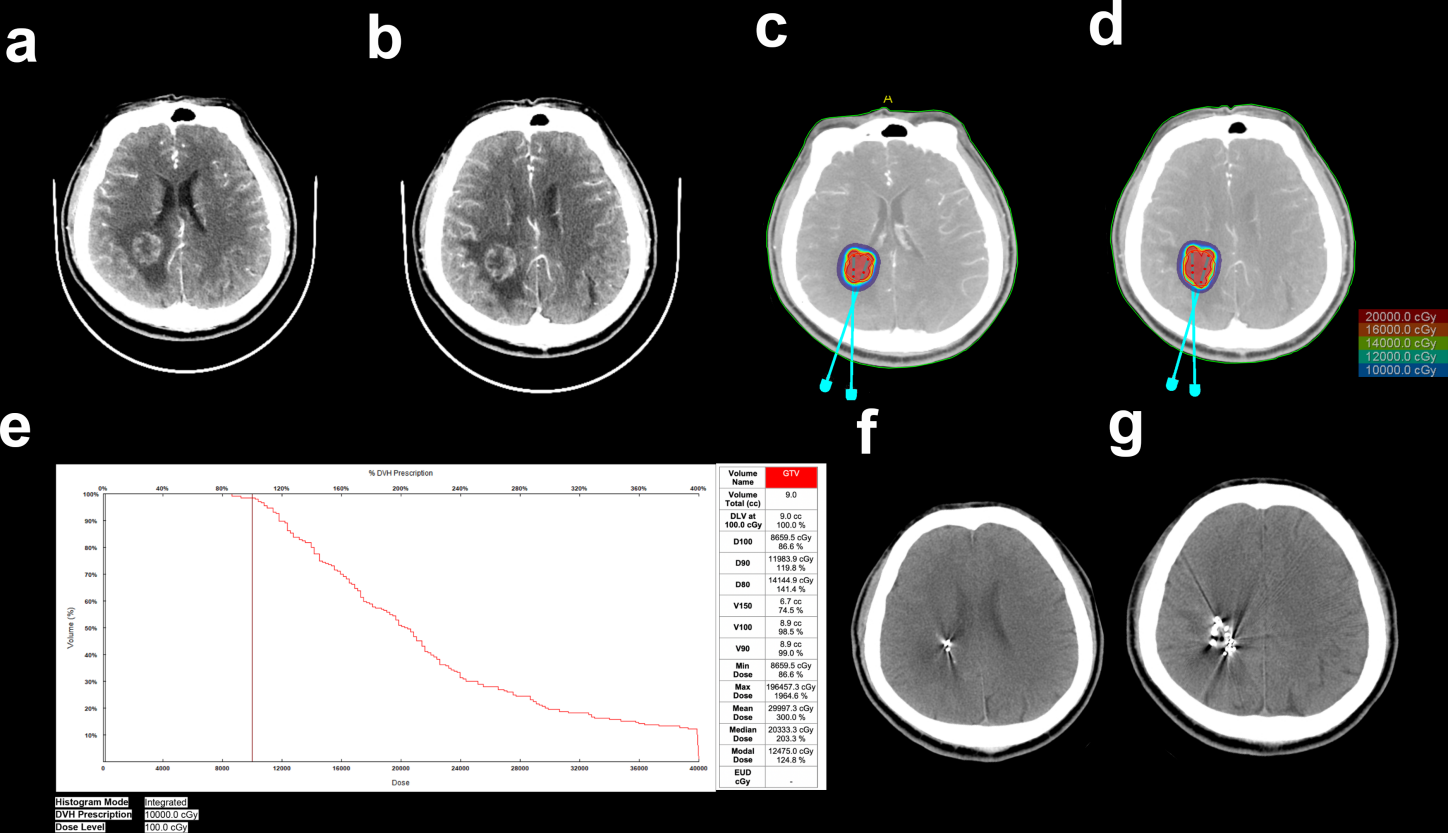
Implantation of ^125^I seeds with the guidance of TPS. **(A, B)** Enhanced CT images of BMs resulting from NSCLC. **(C, D)** Needle paths were designed with TPS. **(E)** Preimplantation dose volume histogram (DVH) of GTV. **(F, G)** CT images of the brain after ^125^I seed implantation.

^125^I seeds were implanted as we previously reported (6, 7). As shown in **Figure 1**, the implantation of ^125^I seeds was performed with the guidance of a treatment planning system (TPS, Beijing Astro Technology Ltd. Co., Beijing, China). Patients were fixed on the CT bed with a vacuum pad. The puncture point was confirmed with a homemade locator. Holes were drilled after general anesthesia according to the preoperative plan. Flat needles were used to implant the ^125^I seeds (0.7 mCi, Model 7711, Beijing Atom and High Technique Industries, Inc., Beijing, China). After the operation, vital signs were monitored, and dehydration medications were given.

### EBRT

**Figure 2:**
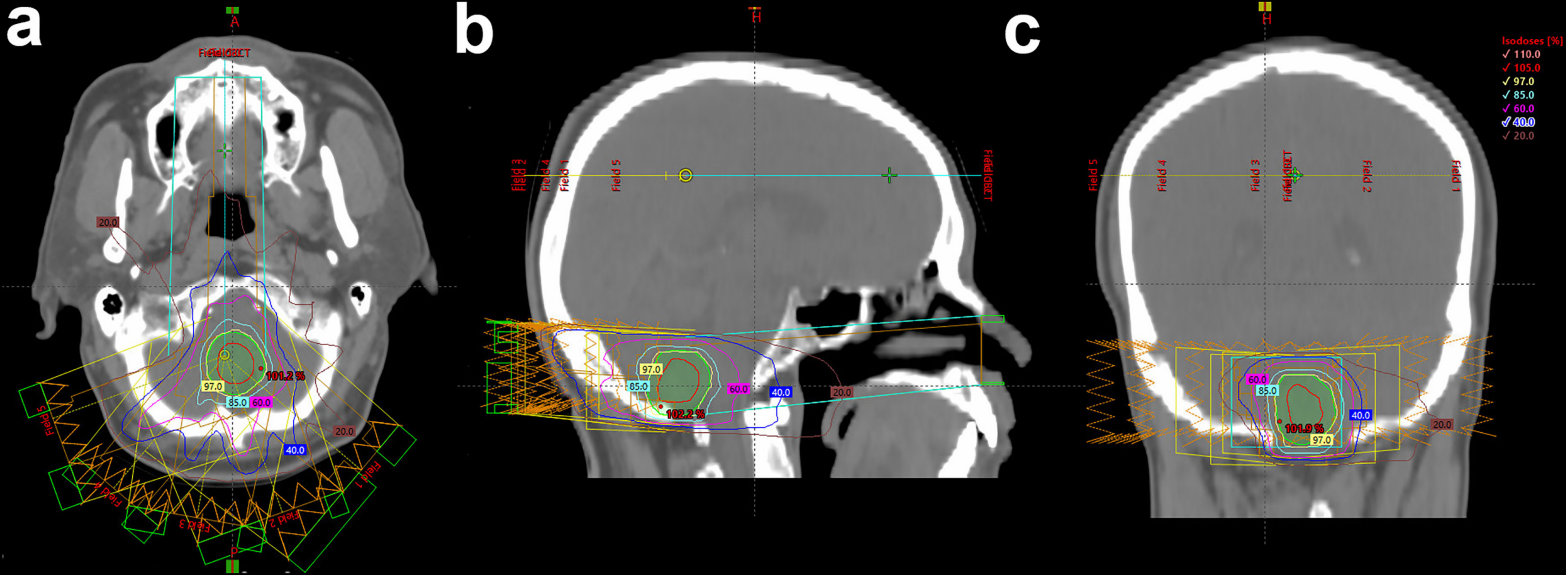
Example of the treatment planning of EBRT. **(A)** Traverse, **(B)** sagittal, and **(C)** coronal views of the treatment planning.

As shown in **Figure 2**, EBRT plans were carried out with a VMAT (RapidArc, Eclipse Treatment Planning System version 13, Varian, Palo Alto, USA) treatment planning system. EBRT planning was carried out with the CT images with a 2-mm slice thickness. According to the standard institutional protocols, the clinical target volumes (CTVs) were delineated. The head was immobilized with a thermoplastic mask. Photon beams which were generated through a linear accelerator and synchrotron were emitted and shaped with ridge filters, double-scattering sheets, multicollimators, and custom-made boluses.

### Study outcomes

Basic characteristics were compared between the two groups. In addition, the 6- and 12-month PFS and the 12- and 24-month OS rates were compared between the two groups. OS was measured from the time of ^125^I implantation or EBRT to the time of death or last follow-up. PFS was calculated from the time of ^125^I implantation or EBRT to the time of tumor recurrence, progression, or death.

### Statistical analysis

The date of the last follow-up was 13 December 2018. SPSS (Version 18.0, IBM, NY, USA) was used to analyze the data. *χ* ^2^ or Fisher’s exact tests were used for categorical variable comparisons, and two-tailed Student’s *t*-tests were used for continuous variable comparisons. Survival curves were evaluated using the Kaplan−Meier method with a log-rank test. For all analyses, a *p*-value <0.05 was considered of significant difference.

## Results

### Patient characteristics

Basic data were compared between the two groups preoperatively. No differences were found in terms of sex, age, Karnofsky performance score (KPS), neurological symptoms, or chemotherapy. In the EBRT group, 5, 42, and 52 BMs were located in the right, left, and both hemispheres, respectively. In the ^125^I group, 32, 21, and 6 BMs were located in the right, left, and both hemispheres (*p* < 0.001), respectively. The ratio of patients with BM diameters greater than 3 cm in the ^125^I group was greater than that in the EBRT group (*p* < 0.001). The median diameter of the tumor was 2 cm (range, 0.6–4.8 cm) in the EBRT group and 3 cm in the ^125^I group (range, 1.5–5 cm). The median prescribed dose (PD) was 41.3 Gy in the EBRT group and 93.5 Gy in the ^125^I group. The differences were significant (*p* < 0.001) (**Table 1**). Brain stem was recognized as the organ at risk. The maximum tolerated dose is 79.6 Gy. Thus, the BED and EQD2 were calculated according to the following formulae (8): BED = *D*[1 + R0/(*μ* + *λ*) (*α*/*β*)] = 83.3 Gy; EQD2 = BED/[1 + 2/(*α*/*β*)] = 50.0 Gy.

**Table 1:** Characteristics of NSCLC patients with BMs in the two groups.

Characteristic	EBRT	^125^I	*p*-value
Gender			0.284
Male	59	30	
Female	40	29	
Age			0.366
≥60	43	30	
<60	56	29	
KPS			0.117
≥90	39	16	
<90	60	43	
Neurological symptoms			0.034
Yes	69	50	
No	30	9	
Tumor location			<0.001
Right	5	32	
Left	42	21	
Both	52	6	
Diameter (cm)			
≥3	24	35	<0.001
<3	75	24	
Median (range)	2 (0.6–4.8)	3 (1.5–5)	
Time between diagnosis and treatment (months)			
Median (range)	2 (0.5–5.5)	2 (0.5–6.5)	
Chemotherapy			0.126
Yes	95	53	
No	4	6	
Dose (Gy)			<0.001
Mean (range)	41.3 (22.8–60)	93.5 (80–120)	

EBRT, external beam radiotherapy; KPS, Karnofsky performance score.

### Postoperative complications

No fatal complications occurred after treatment. No radiation-related necrosis was found in either of the groups. Three patients in the ^125^I group suffered a minor cerebral hemorrhage during the operation. Edema was exacerbated in 15 and 2 patients in the EBRT and ^125^I groups, respectively. No severe neurological symptoms or infections occurred during the therapies.

### Analysis of survival

**Figure 3:**
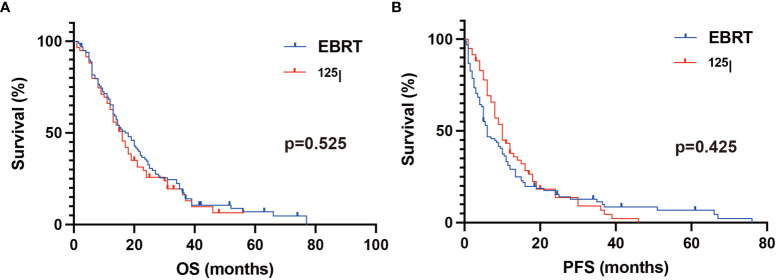
Kaplan–Meier analysis of OS and PFS. **(A)** The median OS was 18 and 16 months in the EBRT and ^125^I groups, respectively. The log-rank test showed no statistical differences between the two groups (*p* = 0.525). **(B)** The median PFS in the ^125^I and EBRT groups was 10 and 6 months, respectively. No significant difference was found between the groups with a log-rank test (*p* = 0.425).

As shown in **Table 2**, the 6-month PFS rates were 51.5% and 76.3% in the EBRT and ^125^I groups, respectively. The differences were significant (*p* = 0.002). The 12-month PFS rates were 30.3% and 40.0% in the EBRT and ^125^I groups, respectively. No significant differences were found (*p* = 0.184). The 12-month OS rates were 66.1% and 67.7% in the EBRT and ^125^I groups, respectively (*p* = 0.839). The 24-month OS rates were 35.4% and 27.1% in the EBRT and ^125^I groups, respectively (*p* = 0.284). The median PFS was 6 and 10 months in the EBRT and ^125^I groups, respectively (*p* = 0.425) (**Figure 3**). The median OS was 18 and 16 months in the EBRT and ^125^I groups, respectively. The log-rank test indicated that no differences were found between OS in the two groups (*p* = 0.525) (**Figure 3**).

**Table 2:** PFS and OS control rate at different time points.

Rate	^125^I (%)	EBRT (%)	*p*-value^a^
6-month PFS	76.3	51.5	0.002
12-month PFS	40.7	30.3	0.184
12-month OS	67.7	66.1	0.839
24-month OS	27.1	35.4	0.284

a Data were compared with the χ^2^ tests.

## Discussion

The present study indicated that both external beam radiotherapy and ^125^I brachytherapy resulted in excellent local control of the disease. The retrospective study suggested that both ^125^I brachytherapy and EBRT are effective therapeutic choices for NSCLC patients with BMs. The ^125^I group had a higher 6-month PFS control rate than the EBRT group.

The prognosis of lung cancer patients with BMs is poor (9). As shown in a Swedish cohort study, the median survival time of lung cancer patients with BMs was only 2.5 months, and the 24-month OS rate was only 10.4% (10). Surgical resection is the best option for patients with single-brain metastasis. Surgery provides a debulking of the mass effect, an effective local control, and tissue for diagnosis. Numerous studies have reported the efficacy of surgical resection for brain metastasis. Nakagawa et al. (11) reported that the overall mean survival was 11.6 months, and the 1-year survival rate was 24% in 89 NSCLC patients with brain metastasis (12). However, surgery was not an option for patients with a large tumor volume in a limited location or in a poor medical condition. For those patients, EBRT is the best option. A series of studies confirmed the therapeutic safety and efficacy of EBRT (9). In recent years, ^125^I brachytherapy has been widely used for various tumors, including prostate cancer, lung cancer, pancreatic cancer, esophageal cancer, colorectal cancer, cervical cancer, head and neck cancer, and liver cancer (13–19).

In the present study, we compared ^125^I brachytherapy with EBRT for NSCLC patients with BMs in whom surgery was not an option. According to the K-M analysis with a log-rank test, there were no statistically significant differences between the two groups in terms of the median OS and PFS. The results indicated that ^125^I brachytherapy could be chosen as an alternative treatment to EBRT for NSCLC patients with BMs. Interestingly, when we compared the 6-month PFS rate between the two groups, ^125^I brachytherapy showed better results than EBRT. The 6-month PFS rates were 51.5% and 76.3% in the EBRT and ^125^I groups, respectively. These results may support that ^125^I brachytherapy was better than EBRT at achieving 6-month local control for NSCLC patients with BMs. Because patients were mostly afraid of radiation damage to normal tissue, the increase in PD was limited during EBRT. ^125^I seeds were implanted within the tumors, and the half-value layers of the seeds in the soft tissue were 1.7 cm. Thus, for BM treatment, the mean PD of the ^125^I seeds was elevated to an average of 93.5 Gy in this study. No severe radiation damage was found. The underlying reason could be that the ^125^I seeds were implanted within the tumor, continuously releasing low-dose γ-rays, which is different from EBRT. The half-life period of ^125^I is 60.2 days (11), and the duration of the therapy is generally considered to be 6 to 8 months, which may partly explain why the 6-month PFS rate was higher in the ^125^I group than in the EBRT group. No significant differences were found when comparing the 12-month PFS rate with the 12- and 24-month OS rates. These results indicated that repeated ^125^I implantation might be needed to obtain a higher 6-month PFS rate.

However, ^125^I seed implantation is a minimally invasive treatment compared with surgical resection. Edema, bleeding, and infection sometimes occur. The complications are typically not severe and can be controlled after conservative treatment. Safety has been proven in several studies (5, 15, 16). No fetal complications were found in either of the groups. Although previous studies reported radiation-induced normal brain tissue necrosis around the lesion during radiation therapy, the risk of radionecrosis is approximately 10%, 15%, or 20% for patients with brain metastases who received single-fraction stereotactic radiosurgery. The volume of the tissues receiving 12 Gy was 5, 10, or >15 cm^3^, respectively (20, 21), while no radiation-related necrosis was found in the present study even with a higher PD in the ^125^I group. As we previously reported, flat needles were used during ^125^I implantation (6, 7). However, bleeding still occurred in 3 patients in the ^125^I group during the operation. Edema was worsened in 2 patients and 15 patients in the EBRT group and the ^125^I group, respectively, which was relieved after routine dehydration medication.

Patients received EBRT Monday–Friday each week for approximately 4–6 weeks. For patients who received ^125^I implantation, the therapy was mostly completed at one time, and patients were discharged from the hospital approximately 3–5 days after the operation. The patients made a one-time payment of approximately $3,000 to $5,000 for EBRT or ^125^I therapy. Although ^125^I is an invasive therapy, it is more convenient for patients than EBRT.

In the present study, we compared ^125^I brachytherapy with EBRT for the treatment of BMs in patients with NSCLC. No significant differences were found between the two groups, except for the 6-month PFS rate. The safety of ^125^I brachytherapy was confirmed. The results indicated that for NSCLC patients with BMs unsuitable for surgery, ^125^I brachytherapy was a safe and effective therapy choice.

## Data availability statement

The data analyzed in this study are subject to the following licenses/restrictions: The dataset is not publicly available, but it can be obtained under reasonable request from the authors. Requests to access these datasets should be directed to Xiaokun Hu, huxiaokun770@163.com.

## Ethics statement

The studies involving human participants were reviewed and approved by The Affilated Hospital of Qingdao University. Written informed consent for participation was not required for this study in accordance with the national legislation and the institutional requirements.

## Author contributions

CW and LY conducted all the experiments, integrated the data, edited the figures, and wrote the manuscript. WZ, SL, TX, and HJ provided essential assistance. XH, MH, and HL directed this study, designed the research, and gave key advice. All authors contributed to the article and approved the submitted version.
